# Understanding the information needs of women with rheumatoid arthritis concerning pregnancy, post-natal care and early parenting: A mixed-methods study

**DOI:** 10.1186/s12891-015-0657-4

**Published:** 2015-08-19

**Authors:** Ilana N. Ackerman, Joanne E. Jordan, Sharon Van Doornum, Margaret Ricardo, Andrew M. Briggs

**Affiliations:** Melbourne EpiCentre, The University of Melbourne and Melbourne Health, 7East, Royal Melbourne Hospital, Parkville, VIC 3050 Australia; HealthSense (Aust) Pty Ltd, Melbourne, Australia; Arthritis and Osteoporosis Victoria, Melbourne, Australia; School of Physiotherapy and Exercise Science, Curtin University, Perth, Australia

## Abstract

**Background:**

Although women with rheumatoid arthritis (RA) face a number of challenges in negotiating the journey to parenthood, no studies have explored the information needs of women with RA in relation to their childbearing years. This study aimed to determine the need for (and preferred mode/s of delivery of) information regarding pregnancy, post-natal care and early parenting among women with RA.

**Methods:**

Interviews and focus groups were conducted with 27 women with RA who were pregnant in the last 5 years, currently pregnant or planning pregnancy. Verbatim transcripts were analysed using both inductive and deductive approaches. Two validated instruments were used to quantify information needs and preferences: the Educational Needs Assessment Tool (ENAT, range 0-156, higher scores indicate higher educational needs) and the Autonomy Preference Index (API, range 0-100, higher scores indicate stronger preferences).

**Results:**

Lack of information about medication safety, access to physical/emotional support services and practical strategies for coping with daily challenges related to parenting were the most prominent of the six key themes identified. Rheumatologists were the primary source for information regarding treatment decisions while arthritis consumer organisations were perceived as critical ‘resource hubs’. There was strong preference for information delivered electronically, especially among rural participants. Quantitative outcomes supported the qualitative findings; on average, participants reported high educational needs (mean ENAT score 97.2, SD 30.8) and API scores indicated that desire for information (mean 89.8, SD 5.6) was greater than the need for involvement in treatment decision-making (mean 68.4, SD 8.2).

**Conclusions:**

Many women with RA struggle to find adequate information on pregnancy planning, pregnancy and early parenting in relation to their chronic condition, and there is a clear need to develop accessible information that is consumer-focused and evidence-based. Although most participants trusted their rheumatologist as their primary information source, there was consistent demand for more information, particularly regarding the safety of RA medications during pregnancy and breastfeeding, and the importance of learning from other women’s personal experiences was strongly emphasised.

**Electronic supplementary material:**

The online version of this article (doi:10.1186/s12891-015-0657-4) contains supplementary material, which is available to authorized users.

## Background

Women of reproductive age living with rheumatoid arthritis (RA) face a number of challenges and decisions while negotiating the journey to parenthood, due to disease-related functional limitations and the potential side-effects of RA medications [1, 2]. As some medications used to limit disease activity and progression are contraindicated during pregnancy and breastfeeding, careful medical planning is required to stabilise disease activity prior to conception and modify medication regimens [3, 4]. Disease activity may improve during pregnancy; however, this is variable [5] and many women experience post-partum flares [6] which impact on their ability to care for their infant, their family and themselves. The pertinent issues encountered by women with RA were recently highlighted in an editorial on RA management and pregnancy [7]. Qualitative research from the United States (US) has also shown that RA can impact significantly on family planning decisions, with women raising valid concerns about the impact of medications on their unborn baby, the heritability of RA and their ability to physically care for a child [8]. The need for accurate information to support women with RA through this important stage of their lives is clear.

While previous studies have identified substantial information needs among people with RA, research has focused primarily on issues related to treatment options and side effects [9, 10] and the accessibility of RA information [11]. Research involving people with RA from the United Kingdom and the US has shown that the need for information was greatest among women [9, 10]. However, no studies have explored the information needs of women with RA during their childbearing years, leaving an important gap in the literature and ultimately, in policies and programmes. This study aimed to determine the information needs of women with RA concerning pregnancy, post-natal care and early parenting, specifically:

- What type of information (in terms of scope and content) do women with RA seek in relation to pregnancy, post-natal care and early parenting?
- In what format and through what channels should this information be available?
- What is the role of consumer-based arthritis organisations in this context?

## Methods

### Study design

Mixed-methods design incorporating two consecutive qualitative data collection phases (individual telephone interviews followed by focus groups) and quantitative data collection. Independent participant samples were used for each qualitative phase. The study was approved by the University of Melbourne Human Research Ethics Committee.

### Participants and recruitment procedures

The study was advertised through arthritis consumer organisations and peer support groups across Australia and via rheumatologists, obstetricians and maternal and child health nurses in metropolitan Melbourne. To be eligible to participate, women were required to:

- be aged 18–45 years;
- have been diagnosed with RA by a rheumatologist;
- be on disease-modifying antirheumatic drugs (DMARDs), except if pregnant or planning to conceive; and
- have been pregnant within the last 5 years, be currently pregnant, or planning to become pregnant in the next 5 years.

**Fig. 1:**
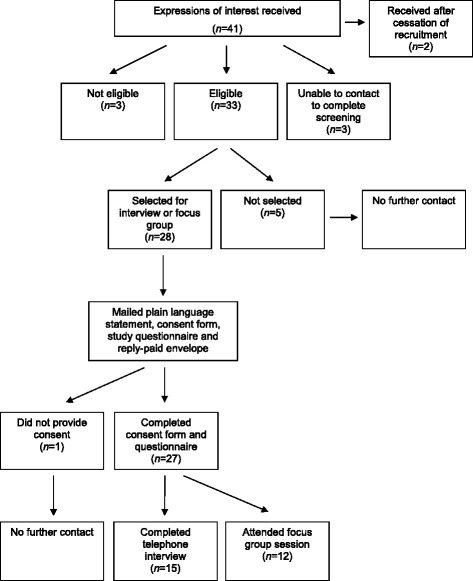
Overview of recruitment and study procedures

Figure 1 provides an overview of the screening and recruitment processes. Interested women were invited to contact a project officer who screened for eligibility. For the interview and focus group phases, maximum heterogeneity sampling was used to generate samples that encompassed a broad range of demographic characteristics [12]. Participants were sampled based on their age, area of residence (urban, regional, remote) and pregnancy status (planning pregnancy, currently pregnant, had been pregnant). Written, informed consent was obtained from all participants.

### Data collection

All data collection was undertaken between November 2013 and January 2014. A mailed questionnaire was used to collect demographic information including country of birth, time since RA diagnosis and employment status. Two validated measures were administered to quantify educational needs and information preferences. The Educational Needs Assessment Tool (ENAT) is a 39-item instrument designed to assess the educational needs of people with rheumatic diseases including RA [13]. It comprises 7 domains including managing pain, movement, feelings, the arthritis process, treatment from health professionals, self-help measures and support from others [14, 15]. Transformed domain scores are summed to produce a total ENAT score ranging from 0 (lowest educational needs) to 156 (highest needs). The Autonomy Preference Index (API) assesses health-related information-seeking and decision-making preferences [16] and has been used previously in RA [10]. The API consists of an Information Seeking Preference Scale (ISPS) and a Decision Making Preference Scale (DMPS). Consistent with earlier research [10], only the first 6 items of the DMPS were used because the remaining 9 items relate to other medical conditions. The ISPS and DMPS scores are each transformed to a 0-100 scale, where higher scores indicate stronger preferences for information-seeking or decision-making, respectively.

#### Telephone interviews

The interview schedule (Additional file 1) was developed by the multidisciplinary research team and 2 women with RA. Semi-structured telephone interviews were conducted by a musculoskeletal researcher experienced in qualitative research methods (INA). Interviews covered perceived gaps in knowledge, sourcing relevant information, further information needs, preferred methods for accessing information, the role of consumer organisations in providing information, and dealing with uncertainty of information.

#### Focus groups

Two focus group sessions were conducted: a face-to-face group involving women living in metropolitan Melbourne and a virtual focus group (using a teleconference facility) for those living in other cities and regional/remote areas across Australia. Virtual focus groups are increasingly popular in qualitative health research and are advocated for overcoming geographic disparity [17, 18]. Data obtained from telephone-based focus groups have been validated against data obtained from face-to-face focus groups [18]. The same schedule was used for the telephone interviews and focus groups.

### Data analysis

Analysis of demographic and questionnaire data was undertaken descriptively using IBM SPSS Statistics version 22. Postcodes were used to classify geographical remoteness, according to Australian criteria [19]. Interview and focus group recordings were transcribed verbatim and transcripts were sent to all participants for verification. The qualitative data were analysed initially by an independent data analyst (JEJ). Data coding was undertaken in two stages, commencing with the interview transcripts. An inductive approach [20] was used to derive key themes from the interview transcripts until no new themes emerged. The focus group transcripts were coded using deductive methods, using the codes derived from the interview data. Where a new topic emerged from the focus group data, a corresponding theme was developed inductively. Each interview and focus group transcript was then reviewed independently by a second researcher (INA) to confirm the themes identified and identify any important omissions. The findings and any discordance were discussed and themes refined to reach consensus, where necessary.

## Results

### Participant characteristics

Twenty-seven women participated in the study: 15 for telephone interviews and 12 for focus groups (*n* = 5 for face-to face, *n* = 7 for virtual). The demographic and clinical characteristics of the participants are summarised in Table 1. The geographic distribution of the sample was broad, with representation from 7 Australian states and territories (Australian Capital Territory: *n* = 1; New South Wales: *n* = 3; Queensland: *n* = 5; South Australia: *n* = 2; Tasmania: *n* = 1, Victoria: *n* = 13; Western Australia: *n* = 2). As shown in Table 1, participants lived in major cities (*n* = 13, 48 %), regional areas (*n* = 12, 44 %) and remote areas (*n* = 2, 7 %).

**Table 1:** Characteristics of study participants

Characteristic	Participants (*n* = 27)
Age (years), median (IQR)	32 (31-36)
Years since diagnosis, median (IQR)	5 (2-13)
Currently under the care of a rheumatologist, *n* (%)	25 (93)
Pregnancy status, *n* (%)	
Pregnant within last 5 years	18 (67)
Currently pregnant	5 (19)
Considering pregnancy in next 5 years^a^	18 (67)
Australian-born, *n* (%)	24 (89)
University education, *n* (%)	19 (70)
Employment status, *n* (%)	
In paid employment	18 (67)
Not working due to parenting responsibilities	8 (30)
Stopped work due to rheumatoid arthritis	1 (4)
Residential location, *n* (%)	
Major city	13 (48)
Regional area	12 (44)
Remote area	2 (7)

IQR: interquartile range

^a^First or subsequent pregnancy

### Educational needs and autonomy preference scores

The mean (SD) total ENAT score was 97.2 (30.8), indicating that, on average, participants reported high educational needs. The ENAT domain scores (Table 2) revealed that the greatest information needs were in relation to the arthritis disease process and treatments from health professionals.

**Table 2:** Educational needs and autonomy preference scores

Outcome measure	Mean (SD)	Range
Educational Needs Assessment Tool (ENAT)		
Managing pain domain	14.2 (4.8)	0.0 - 24.0
Movement domain	11.0 (4.5)	3.1 - 20.0
Feelings domain	10.7 (4.3)	0.0 - 16.0
Disease process domain	19.2 (5.7)	8.9 - 28.0
Treatments from health professionals domain	17.5 (7.0)	2.2 - 28.0
Self-help measures domain	16.0 (6.2)	5.0 - 24.0
Support from others domain	8.7 (3.9)	3.0 - 16.0
Total ENAT score^a^	97.2 (30.8)	27.3 - 156.0
Autonomy Preference Index (API)^b^		
Information seeking preference scale	89.8 (5.6)	77.5 - 100.0
Decision making preference scale	68.4 (8.2)	50.0 - 83.3

^a^Total ENAT score ranges from 0 (lowest educational needs) to 156 (highest educational needs)

^b^API scale scores for each scale range from 0 (low preference for information-seeking or decision-making) to 100 (strongest preference for information-seeking or decision-making)

The Autonomy Preference Index data (Table 2) showed that the mean (SD) score for the DMPS was 68.4 (8.2), with scores ranging from 50.0 to 83.3. The mean score for the ISPS was very high (89.8, SD 5.6), indicating that participants’ need for information exceeded their need for involvement in treatment decision-making.

### Interview and focus group data

Six key themes emerged from the interviews and focus groups, as summarised in Table 3 and outlined in further detail below. The themes are presented to provide a logical understanding of the issues encountered by women with RA. Analysis of the focus group data reinforced the themes identified from the interviews, with some additional subthemes emerging.

**Table 3:** Key emergent themes from the interviews and focus groups

Theme	Description
Lack of specific information regarding the pregnancy and post-natal periods	• Perceived lack of consumer-focused written information relating specifically to RA and pregnancy, the post-natal period and early parenting
	• Perception that the needs of younger people with arthritis are not well addressed
Information needs are dependent on the individual situation	• Difficulty of knowing what specific information will be needed given the variability in how RA can be affected by pregnancy
	• Information needs vary according to maternal stage and educational or professional background
Rheumatologist as the primary information source	• Expressed trust in rheumatologists who provide individuals with most of the information relating to RA and pregnancy
Identified information gaps	• Four main subthemes were identified in relation to information gaps:
	• drug toxicity
	• physical and emotional support services
	• practical tips and strategies to assist in coping with daily challenges
	• information for family, friends and the workplace
Accessible information through electronic formats	• Clear preference for written information which can be made available via electronic formats and updated regularly to maintain relevance
Arthritis consumer organisations as a resource hub	• Role of organisations should be to:
	• collate and provide access to up-to-date, evidence-based information
	• provide a referral system to support services and facilitate peer support groups
	• provide RA-related education and training to upskill health professionals commonly encountered by women during this life stage

#### Key theme 1: Lack of specific information regarding pregnancy and RA

There was general consensus among participants regarding a dearth of consumer-focused information that comprehensively addressed issues encountered by women with RA across the pregnancy continuum:

*“…I got my RA during pregnancy with my first baby, and I’d say the first five years of having RA … I had no idea, information was scarce, I had done millions of Google searches and went to libraries and there was nothing. Nothing about pregnancy, breastfeeding and RA. And even my doctors were really… didn’t seem to know much about what medications were safe, and they’d have to go and ring other doctors, and it was really just like… I thought gee, am I the first person in the entire world to have RA and be pregnant, you know…I think nowadays you can find more information but it is like one of the other ladies said, it’s pulling teeth, it’s not offered up to you…you’ve really got to search hard sometimes, and even just to find another patient who you can talk to is not easy. It’s really hard to find.”*

Several participants referred to a published book [21] which detailed personal experiences of pregnancy with RA as their only real resource. There was also a strong perception that the needs of younger women with arthritis are currently not well addressed, particularly by arthritis consumer organisations, and that arthritis is widely perceived as a condition affecting older people. Several women considered that this misunderstanding was reinforced by images commonly used in arthritis educational materials:

*“I picked up a brochure for my mother which was about…a brochure for carers of people with arthritis, and just so she could get some understanding, but the picture on the front was a really, old wrinkly hand and it was all kind of twisted, and I thought oh, that’s not a good perception. People don’t…and when you tell them you have arthritis they really don’t understand because they think it’s still, yeah, it’s still an old people’s thing.”*

#### Key theme 2: Information needs dependent on individual situation

Participants expressed difficulty knowing what specific information they might need given the uncertainty about their disease activity during pregnancy and following childbirth. Additionally, information needs varied depending on the life stage and educational or professional background. Participants with a health professional or research background had a clearer understanding of what information they needed and reported being more successful in sourcing information. One participant advocated for documenting different pregnancy scenarios and making these available to provide reference for medical practitioners and patients.

#### Key theme 3: Rheumatologist as the primary information source

The majority of telephone interviewees cited their rheumatologist as their primary information source, particularly in relation to medication decision-making. Participants spoke more about trusting their rheumatologist’s judgment rather than wanting to understand detailed information relating to RA medications:

*“Look I trust in my rheumatologist. Sometimes it's, look and I have read up and it scared me a little bit with information, so sometimes I think it's best if I just leave…I don't investigate too much, I just trust in my rheumatologist and if she says it's safe, it's safe.”*

Some participants also sourced information from the internet, including online forums and social media. Focus group participants also expressed trust in their rheumatologist, but regularly sought additional sources of information (primarily from online sources) in order to make informed treatment decisions.

#### Key theme 4: Identified information gaps

Irrespective of pregnancy status, participants clearly identified four distinct areas where further information is required:

- drug toxicity
- access to physical and emotional support services
- RA-related information for families and workplaces
- practical strategies to assist with daily challenges

Of greatest concern to participants was the need for more information regarding the toxicity of RA medications and the possible effects on their unborn or breastfed baby. This related to the safe use of RA medications around conception, pregnancy and breastfeeding and options regarding other medications, alternative therapies or lifestyle modifications.

*“I guess I’d like really clear answers on medications and exactly what they do or don’t affect in terms of pregnancy and breastfeeding and conception. And I know that information is hard to get but I think it’s really important to have…surely there’s a lot of things like diet and exercise and other relaxation techniques, those kind of things, surely can help on the peripheral sort of areas just to minimise your symptoms. And so more information about that would be great.”*

A distinguishing feature of the focus group discussions was the need for more decision-making support, primarily more direct guidance from rheumatologists, when selecting medications for use during pregnancy or breastfeeding. Participants appreciated this was a complex area given variable individual needs, but wanted to better understand the possible range of scenarios and treatment options, the consequences of not taking medications, and different treatments for managing post-partum flares.

Participants also expressed considerable frustration at receiving inconsistent advice and the lack of knowledge regarding RA and its management among nurses, medical practitioners and pharmacists:

*“But what I find frustrating too is when you actually go into your pharmacy to get your medication and then the pharmacist disagrees with what your doctor says, and you have to argue with them to get your pills…you have to always put this argument in your mind, and to everyone else around you that you’re not…you know, you don’t want to harm your baby but you also need to be able to move…and it’s an inner struggle as well as a struggle with everybody else that knows what you’re taking…and between doctors…like rheumatologists have different opinions on what’s safe and what’s not, so it’s really…yeah, frustrating would be the top word I think.”*

Participants expressed a strong need for information about appropriate support services, particularly in the post-natal period. These included physical support services to assist with household tasks, and access to maternal and child health nurses for advice regarding activities of daily living. The majority of participants reported feeling isolated and uncertain about what to expect during the pregnancy and post-natal stages, and perceived that emotional support services (including counselling and peer support groups) would be beneficial. Learning from other women’s experiences was an important source of emotional support, particularly when managing post-partum flares and making difficult decisions such as ceasing breastfeeding in order to re-commence RA medications.

*“…if there were even volunteers on some phone line or something you could ring, but, you know, I can remember going to the maternal health centre and telling them that… you know, with the pain was just getting too bad and I was going to have to go onto these drugs again and I’d have to stop breastfeeding, and we had a very young maternal health nurse at the time and she said well you just have to decide whether you’re going to be selfish and choose yourself over your child…you know that's a hard decision, and yeah, if you had a support person that you could sort of lean on I think that would be really helpful.”*

Participants consistently expressed a desire for practical tips and strategies to assist them, particularly during the post-natal period. While there was some interest in learning about the latest research findings, there was a clear preference for pragmatic information from peers to enable women to cope with the day-to-day challenges of caring for a young baby (for example, bathing, dressing and feeding tasks). Focus group participants also perceived a need for information about assistive devices that could facilitate daily activities, such as using a baby car seat.

*“Some practical information as to how to do certain daily tasks without the use of certain limbs. So, you know, if my hands are so swollen that I can't dress myself, how do I pick up my baby using my forearms, how do I…how can I carry my baby to minimise the impact on my arms and shoulders and back and wrists etc. Clothing to put the baby in…that was a big thing for me, you know, most baby clothes have…stupid press-studs, yeah, which were just the bane of my life back then. So and I managed to find clothing that was much easier for me to get on and off the baby - so that was, you know, that was a big thing. And, you know, even just…every day household tasks, you know, tips as to how to, you know, reduce your fatigue and cut down on your household duties and to…minimise your energy output.”*

A new subtheme to emerge from the focus groups was the need for information to be made available for family, friends and work colleagues, to improve their understanding of the challenges faced and garner support.

*“Just something that I think would be really helpful is also some information that you can give to your close…your partner, support people like your parents or your siblings or whatever, because I think a lot of women, especially as new mums, find it hard to reach out for help or support. But if there was something that you could actually just go, oh yeah, this is what my rheumatologist gave me to give to you and it said something like, yeah, this is what they’re actually…this is what she’s going through and…these here are some of the ways that you can help or this is some of the ways that this person needs some help, that would be really beneficial.”*

#### Key theme 5: Accessible information through electronic formats

While participants preferred written information, there was variation among women as to how this information should be delivered. Some participants, particularly those in rural or remote settings, expressed a strong preference for electronic information (via email or online sources) that could be easily retrieved, while others preferred information packs from health professionals or arthritis consumer organisations. Focus group participants emphasised the need for regular review of materials to ensure their relevance.

#### Key theme 6: Arthritis consumer organisations as a resource hub

Although many participants had contacted their local arthritis consumer organisation, some indicated they were unable to obtain the information they were seeking. Interviewees and focus group participants identified three main roles for arthritis organisations:

1. as central resource hubs which collate and provide accessible, up-to-date, evidence-based information from local and international sources;
2. to provide referral systems for directing consumers to relevant support services (including facilitating peer support groups); and
3. to provide disease-specific education and training for health professionals who commonly encounter women with RA during the pregnancy and post-natal periods.

*“…I think we need to be realistic and rheumatologists don’t have time to go through all the other components that need to be addressed. So I think consumer organisations need to take on a huge role and get lots and lots of knowledge about it and they need to be - it needs to be - an area that there are courses run all the time, the information sessions run all the time, the written information sent out, there's telephone counselling where someone can ring and talk, you know, talk about the issues. I think it needs to become one of those areas that is a standard part of the service that they offer, cause I think it's really, really important.”*

## Discussion

Using rigorous qualitative methods, this study has generated new and detailed information about the specific information needs of women with RA during their reproductive years. The data clearly demonstrate that women struggle to find consumer-focused, accessible information on pregnancy planning, pregnancy and early parenting in relation to their chronic condition. Although most participants trusted their rheumatologist as their primary source, there was consistent demand for more comprehensive information and the importance of learning from women’s personal experiences was strongly emphasised. In addition to disease-oriented information, the need for information about available physical and emotional support services and improved advocacy about the impact of RA was evident.

Our quantitative data showed that participants had a strong preference for information-seeking, which echoes the interview and focus group themes. In contrast, the need for involvement in decision-making was substantially lower, and this is consistent with research involving 600 people with RA in the United Kingdom (UK) [10]. Our quantitative findings closely reflect the qualitative data, in that women strongly desired further information yet placed great trust in their treating rheumatologist, particularly regarding medication decisions. A recent multi-national survey of women with RA also found that 91 % of participants believed their rheumatologist was capable of helping with treatment decision-making during pregnancy [22]. The relatively low decision-making scores in our study plus the desire for more guidance with medication decision-making, as raised by several participants, could reflect the perceived lack of adequate information which then increases the reliance on medical specialists. Mean domain and total scores for the ENAT instrument were comparable to those reported for 98 women with RA in a recent Austrian study [23], indicating that our study participants expressed similar educational needs in relation to managing pain, medication use and obtaining support. Similar to the findings of Dragoi et al. [23], the greatest educational needs centred on the disease process and treatments.

While consumer-focused information relating to RA is publically available, participants considered that materials specific to pregnancy and post-natal care are lacking. Our data indicate that information targeted at women with RA needs to comprehensively cover issues across the pregnancy continuum and be segmented so that women can access information relevant to their stage and experience. This fits well with recent European League Against Rheumatism (EULAR) recommendations about the importance of needs-based patient education that can be accessed over the disease course and at different life stages [24]. Digital modes would be the most efficient way of delivering information, particularly for women in non-metropolitan areas [25], and could be readily updated as new therapies or resources become available. For example, Interactive Health Communication Applications use contemporary electronic formats to provide patient information for chronic conditions, with demonstrated positive effects on knowledge, social support and clinical outcomes [26]. A number of countries have already developed information portals for women with rheumatic diseases, although it is evident that these resources are not being sufficiently disseminated to women with RA in Australia (or their healthcare providers), leaving women with unmet information needs. For example, the Arthritis Foundation in the US offers several webpages relating to rheumatic diseases and pregnancy [27], as does Arthritis Research UK [28] and the National Rheumatoid Arthritis Society in the UK [29]. While study participants desired evidence-based information, they also sought practical information from peers regarding functional and coping strategies. This mirrors the desire of health professionals to receive information from key opinion leaders [30] and aligns with contemporary knowledge translation approaches which combine simplified, evidence-based messages with peer-to-peer information exchange, often through narratives [31]. Booklets containing practical information for women with RA are distributed to patients in countries such as Norway [32], and a version tailored to meet the needs of Australian women would be a valuable resource.

Our findings have several implications for practice and policy and we have developed detailed recommendations to assist arthritis organisations and health professionals (Table 4). While these focus on improving information provision for women with RA, a key recommendation involves developing disease-specific training programs to upskill health professionals commonly encountered during the pregnancy and post-natal periods. Increasing awareness of pregnancy and post-natal issues across the range of healthcare providers is also essential. Given the frustration described by many participants, developing health workforce capacity to deliver accurate, consistent information represents an important area for development. This is particularly relevant to allied health professionals, pharmacists and nurses where RA knowledge and skills gaps have been reported [33–35]. It is also in line with recent EULAR recommendations that highlight the need for providers of patient education in inflammatory arthritis to have access to specific training in order to develop and maintain their knowledge and skills [24]. Arthritis organisations also have an important role to play, from acting as ‘resource hubs’ to facilitating peer support (for example, by organising or initiating groups that enable women with RA to share their personal experiences of pregnancy). Establishing cross-discipline consensus concerning appropriate messages for women with RA regarding medication safety is an important avenue for future research that we are now pursuing.

**Table 4:** Key recommendations for addressing identified knowledge and service gaps

1. Develop an online resource hub that could be administered by arthritis consumer organisations, comprising:
• up-to-date, evidence-based information on medication safety for conception, pregnancy and breastfeeding
• practical strategies for managing activities of daily living with a young baby
• information on specific aids and appliances to facilitate activities of daily living during the post-natal period
• detailed information on RA for family, friends, employers and colleagues including specific challenges during the pregnancy and post-natal periods and suggestions for supporting women with RA
• a list of obstetricians with specific experience or special interest in planning and managing pregnancies in women with RA
• a list of available support services including home help, counselling services, medication safety information services and government financial support options
• resources for clinicians who may encounter women with RA during the pregnancy and post-natal stages
2. Offer multiple information formats to comprehensively meet the needs of women with RA, for example:
• online educational materials
• seminars/workshops incorporating specialist health professionals and women with RA who can share first-hand experiences and demonstrate practical strategies
• written education materials that can be mailed or emailed
• telephone counselling services staffed by trained health professionals
3. Facilitate online peer-support/mentoring programs to enable women to share personal experiences
• consider the development of ‘outreach’ programs involving home visits by health professionals and/or peers during the early parenting period
4. Promote the role of arthritis consumer organisations and available resources to relevant health professionals
5. Develop disease-specific training programs to upskill midwives, maternal and child health nurses and allied health professionals
6. Raise awareness of RA as a form of arthritis which affects younger women of reproductive age and use appropriate images in publications and online materials to reflect this demographic

This study has several strengths, including the national sample that comprised participants from metropolitan, regional and remote areas. Dual data collection phases were used to minimise bias and achieve a comprehensive set of themes [36]. Additionally, our mixed-methods approach used validated quantitative tools to assess educational needs and preferences, enabling comparison with international datasets and corroboration of qualitative data. However, we acknowledge that this research did not capture the views of family members or health professionals, nor the perspectives of women with other inflammatory arthritis. This study focused on RA to minimise disease heterogeneity among participants.

## Conclusions

Specific knowledge and service gaps exist in relation to RA and the pregnancy and postnatal continuum. There is a pressing need to develop accessible, consumer-focused information, including resources that enable learning from other women’s experiences, to support women with RA during pregnancy and beyond.

## Additional file

Additional file 1:
**Schedule for interviews and focus groups.** (PDF 19 kb)
