# Supplementary material for: Understanding the information needs of women with rheumatoid arthritis concerning pregnancy, post-natal care and early parenting: A mixed-methods study
Source: BMC Musculoskelet Disord. 2015 Aug 19;16:194. doi: 10.1186/s12891-015-0657-4 (PMC4545539; doi:10.1186/s12891-015-0657-4)
Supplement: Additional file 1: — Schedule for interviews and focus groups. (PDF 19 kb) [file 12891_2015_657_MOESM1_ESM.pdf]

Additional file 1: Schedule for interviews and focus groups

1. Perceived gaps in individual knowledge

   Suggested prompts:
   - Can you tell me about your current level of knowledge about pregnancy, postnatal care and early parenting, in relation to your RA?
   - Do you think there are any gaps in your current knowledge about RA in relation to pregnancy, postnatal care and early parenting?

2. Finding information about pregnancy, postnatal care and early parenting, in relation to RA

   Suggested prompts:
   - How have you tried to find information about pregnancy, postnatal care and early parenting, in relation to your RA?
   - How successful have you been in finding information about pregnancy, postnatal care and early parenting, in relation to your RA?
   - Can you tell me about areas that you think are currently covered well, either by your doctor or other information sources?
   - Can you tell me about areas that you think are not covered well, either by your doctor or other information sources?

3. Further information needs

   Suggested prompts:
   - Thinking about your RA and pregnancy, can you give some specific examples of what information would be helpful for you?
   - Thinking about your RA and postnatal care, can you give some specific examples of what information would be helpful for you?
   - Thinking about your RA and early parenting, can you give some specific examples of what information would be helpful for you?
   - What would be more helpful to you - information about the latest research evidence or information about practical strategies and tips? Can you explain your decision?

4. Preferred format and channels for accessing information

   Suggested prompts:
   - How would you prefer information about pregnancy, postnatal care and early parenting, in relation to your RA, to be provided to you?
   - In what format would you prefer this information to be made available?

5. Role of consumer organisations, such as Arthritis Victoria, in providing information

   Suggested prompt:
   - What do you see as the role of consumer organisations, such as Arthritis and Osteoporosis Victoria, in providing information about pregnancy, postnatal care and early parenting in relation to your RA?

6. Dealing with uncertainty of information

Suggested prompts

- How do you deal with the lack of good evidence relating to the safety of some rheumatoid arthritis medications, with regard to pregnancy and breastfeeding?
- How do you decide which information to trust or use to make your treatment decisions?
